# The health policy processes section: scope and future

**DOI:** 10.1093/heapol/czag076

**Published:** 2026-08-18

**Authors:** Sudha Ramani, Yusra Ribhi Shawar, Eleanor Beth Whyle

**Affiliations:** Independent Consultant, Mumbai; Bloomberg School of Public Health and School of Advanced International Studies, Johns Hopkins University, 615 N Wolfe St, Baltimore, MD 21205, United States; School of Public Health: Health Policy and Systems Division, University of Cape Town, Barnard Fuller Building, Anzio Rd, Observatory, Cape Town 7935, South Africa

Health Policy and Planning (HPP) is one of the few journals in the field of public and global health that has a dedicated Section on health policy processes in low-and middle-income countries (LMICs). In this editorial, we explain the rationale behind this Section, highlight the value it brings to scholarship in this field, and point to the kind of papers we will welcome henceforth.

Health policies refer to decisions, plans and actions taken to achieve specific health and healthcare goals within a society (WHO 2018 cited in Gilson et al. 2018). We take these goals to extend beyond the delivery of health services to include social, political and economic determinants of population health. We view policies as more than merely neutral or technical instruments for achieving these goals; with an understanding that policies are products of the social, economic and political contexts that shape their development and implementation (Walt and Gilson 1994).

The term ‘policy processes’ in the Section title encompasses the entire policy cycle—including agenda-setting, formulation, adoption, implementation, and evaluation of policies—recognizing the interconnectedness between each stage and how they collectively shape policy outcomes. The study of policy processes can, therefore, include research on how health systems respond to policy interventions, how systemic factors constrain or enable policy change, and how policies are implemented in complex systems—which creates some overlap with other sections of the journal.

The purview of this Section differs from our sister Section “Health Systems Research,” as we are primarily concerned with the politics of policy processes in health systems, rather than with their features, governance arrangements, and functioning. Likewise, our focus differs from that of the “Implementation Research and Evaluation” section, in that our focus is not only on the ground-level workings of policies but also on relationships and power dynamics between different actors; contestation over ideas during implementation; and the workings of formal and informal institutions (Gilson et al. 2018). In keeping with the broader field of health policy analysis (Gilson and Raphaely 2008, Whyle et al. 2026), research in this Section is grounded in interdisciplinary theories and analytical frameworks from political sciences and other social sciences.

## The need to study health policy processes in LMICs

Although scholarly work on policy processes in LMICs is increasing, much of the empirical research to date about these processes, and many of the theories that dominate the field, have been based on HIC policy experiences (Gilson and Raphaely 2008, Walt et al. 2008, Parkhurst 2023, Whyle et al. 2026). Research on LMIC policy processes needs to account for the distinct contexts in LMICs, including resource constraints, social inequities, political histories, institutions, and cultural identities. In addition, in some LMICs, global health actors including international donors play key roles in how policies develop and unfold in practice, adding a new transnational layer of complexity for policy analysis in these contexts (Sparkes et al. 2024). Thus, there is a pressing need for policy research from LMICs that is contextually grounded and may contribute to the refinement of broader health policy theory.

At present, much of the research that is being produced on policy processes in general appears in journals from the political science, public administration and development studies space. These journals, while rich in discipline-oriented theory and insights, do not foreground the particularities of public health challenges and health policy processes. In addition, these journals may be less accessible to health policymakers and practitioners from LMICs. All these points indicate a clear need for more academic spaces within the health field that focus on policy processes in LMICs.

## What we have published so far and what we are looking for

The collection of articles published in the Section in recent years spans a diverse range of thematic areas, policy stages and processes, and methodologies. In the sub-sections that follow, we reflect some of the thematic ideas, methodological approaches, and theoretical contributions that have been valued within the section. We also offer some directions on the kind of articles that would be of future interest in this section.

### Thematic areas of importance

Recent papers in this section have focused on emerging policy issues in LMICs including those related to specific illnesses, disease conditions and emerging health threats. We published a series on COVID-19 policy responses (Gilson et al. 2020) and other important articles on how ideas and institutions shape policy response to infectious diseases such as malaria (see Parkhurst et al. 2021, Webster et al. 2021). In addition, we have published articles that seek to explain the priority given to infectious diseases in the global health agenda (Smith et al. 2024). We have also published a number of impactful articles on non-communicable disease policy, including on ‘sugar taxes’ (see Carriedo et al. 2020, Mulcahy et al. 2022), tobacco control (see Lencucha et al. 2020, Udokanma et al. 2021, Akin-Onitolo and Hawkins 2022) and alcohol regulation (see Hargovan et al. 2024). In addition, the Section has been contributing to an emerging body of work on mental health policy (see Iemmi 2022, Sabey 2022, Ionescu et al. 2024, Tele et al. 2025).

The section is also particularly strong in publishing analyses of health financing reforms, including UHC and social or national health insurance programs (see Wireko et al. 2020, Novignon et al. 2021, Srivastava et al. 2023, Ridde et al. 2024), as well as donor dependency and transition (see our supplement on rethinking external assistance: https://academic.oup.com/heapol/issue/39/Supplement_1). A particularly robust thematic area explores the regulation and influence of private actors (see. Cross et al. 2017, Hoe et al. 2021).

In future, we see the need for more papers that examine contested policy arenas involving multiple actors and sectors. For example—NCDs and mental health policy reforms require long-horizon, multi-sector coalitions that can withstand stigma and industry lobbying, and anti-microbial resistance demands “One Health” coordination and new surveillance and enforcement capacities. These complex challenges are particularly well suited to policy analysis, as understanding them requires paying close attention to power, politics and contestation. We also welcome further scholarship that builds on previously published series and supplements, and submissions on emerging and cross-cutting themes that have received relatively less attention in our section thus far; for example, the emerging role and influence of artificial intelligence in LMIC policy processes.

### Articles across policy stages

The section accepts articles on, and has contributed to scholarship on, all stages of the policy process. Articles have examined, for example, how a country’s historical legacy shapes what is seen as a policy problem and its solutions (e.g. de M Pontes and Santos 2020), why certain policies gain priority in LMICs while others do not (e.g. Shawar and Crane 2017), why even well-designed policies face implementation challenges and sometimes fail in practice (such as Jassat et al. 2025 on implementation of a decentralized drug-resistance tuberculosis policy in South Africa).

We also welcome both retrospective policy analyses (including those examining policy processes using historical methods), and forward-looking or prospective policy analyses, seeking to contribute to policy change. We recognize that history-sensitive analysis of policy processes can give insights into how past choices, colonial legacies and path dependencies continue to influence present-day policies. At the same time, there is a need for real-time, forward-looking analysis done in anticipation of future challenges (Buse 2008), and such analyses are limited in the field (Whyle et al. 2026).

### Methodologies for deeper policy analysis

We intend the Section to make a substantive contribution to methodological evolution in the field, and welcome articles using innovative interpretive and discursive analytic approaches, such as framing analysis (Koon et al. 2016) or the “What's the Problem Represented to Be” approach (Bacchi and Eveline 2010). These approaches allow for deeper interrogation of what policies mean, uncovering the values, assumptions and power relationships reflected within them. For example, Namugumya et al. (2021) present an empirical study of how competing understandings of malnutrition have contributed to inconsistent prioritization of the issue in Uganda.

Further, we are interested in papers that contribute to the development of methodological approaches for policy analysis. Indeed, some of the Section’s most impactful contributions have been methodological, such as those included in the “How to do…or not to do” series (e.g. Sriram et al.’s 2018 “10 best resources on power in health policy and systems in LMICs” and Dalglish et al.’s (2021) “Document analysis in health policy research: the READ approach”). Such articles are frequently assigned as foundational readings in graduate courses and act as on-ramps for new researchers.

### Engagement with theory

We strongly encourage authors to engage with policy analysis theories, frameworks and lenses in their submissions. For example, the Political Economy Analysis framework from Sparkes et al. (2019) has been applied by Novignon et al. (2021) to examine the political pathways that led to the establishment of Ghana’s National Health Insurance Scheme. Similarly, Parashar et al. (2020) used actor interface analysis to explore how power was exercised during the implementation of a free maternal and child health policy in India.

However, theories must not be perceived as rigid ‘straight-jackets,’ but rather as heuristic devices. We also encourage authors to reflect explicitly on adaptations, limitations, and the contextual nuances of the theories they employ—including where concepts travel poorly, require modification, or yield competing explanations. One persistent challenge in this Section is the tension between accessibility and theoretical sophistication. On one hand, deep engagement with theory can help researchers navigate complexity and improve rigor. On the other hand, theories from the social sciences can be perceived as unhelpfully abstract by those working in the applied public health field, and public health researchers not trained as social scientists may be hesitant to draw on theory. We encourage contributions that attempt to strike a balance between theoretical grounding, practical application for LMIC settings and accessibility to a diverse public health audience.

## Final reflections

HPP offers a dedicated space for analysis of policy processes in LMICs. We are proud of our legacy as a dedicated space for LMIC-focused research, and remain committed to encouraging and enabling the publication of LMIC voices.

We believe that this commitment is particularly important in today’s policy environment. Epidemiological transitions, the withdrawal of external aid, the growing influence of commercial actors, and the rise of authoritarian politics are reshaping who sets policy agendas, how coalitions form, and what implementation strategies are feasible in LMICs. These developments require engagement with complex and ‘wicked’ problems that have long been the hallmark of this Section.

While we recognize that *Health Policy and Planning* is read predominantly by academics, our Section on policy processes hopes to be accessible to a wider audience. The journal’s shift to open access is a positive step in this regard. Also, articles published in the journal are already routinely cited in policy documents (https://academic.oup.com/heapol/pages/policy-and-global-impact). We strive to ensure that the key messages of articles in the policy process section are written with policymakers and practitioners in mind. In this way, the Section hopes to contribute both to scholarship and practice in this field.

## Data Availability

Not applicable for this editorial.

## References

[czag076-B1] Akin-Onitolo A, Hawkins B. Framing tobacco control: the case of the Nigerian tobacco tax debates. Health Policy Plan 2022;37:22–32. 10.1093/heapol/czab095 34369574

[czag076-B2] Bacchi C, Eveline J. What’s the problem represented to be? In: Mainstreaming Politics: Gendering Practices and Feminist Theory. University of Adelaide Press, 2010.

[czag076-B3] Buse K . Addressing the theoretical, practical and ethical challenges inherent in prospective health policy analysis. Health Policy Plan 2008;23:351–60. 10.1093/heapol/czn026 18664524

[czag076-B4] Carriedo A, Lock K, Hawkins B. Policy process and non-state actors’ influence on the 2014 Mexican soda tax. Health Policy Plan 2020;35:941–52. 10.1093/heapol/czaa060 32672333

[czag076-B5] Cross HE, Sayedi O, Irani L et al Government stewardship of the for-profit private health sector in Afghanistan. Health Policy Plan 2017;32:338–48. 10.1093/heapol/czw130 27683341 PMC5400060

[czag076-B6] Dalglish SL, Khalid H, McMahon SA. Document analysis in health policy research: the READ approach. Health Policy Plan 2021;35:1424–31. 10.1093/heapol/czaa064 33175972 PMC7886435

[czag076-B7] de M Pontes AL, Santos RV. Health reform and Indigenous health policy in Brazil: contexts, actors and discourses. Health Policy Plan 2020;35:i107–14. 10.1093/heapol/czaa098 33165584 PMC7649663

[czag076-B8] Gilson L, Marchal B, Ayepong I et al What role can health policy and systems research play in supporting responses to COVID-19 that strengthen socially just health systems? Health Policy Plan 2020;35:1231–6. 10.1093/heapol/czaa112 33020815 PMC7665452

[czag076-B9] Gilson L, Orgill M, Shroff ZC. A Health Policy Analysis Reader: the Politics of Policy Change in Low-and Middle-Income Countries. Geneva: Alliance for Health Policy and Systems Research, World Health Organization, 2018.

[czag076-B10] Gilson L, Raphaely N. The terrain of health policy analysis in low and middle income countries: a review of published literature 1994–2007. Health Policy Plan 2008;23:294–307. 10.1093/heapol/czn019 18650209 PMC2515407

[czag076-B11] Hargovan M, London L, Orgill M. The influence of crisis on policy formulation: the case of alcohol regulation in South Africa during COVID-19 (2020–21). Health Policy Plan 2024;39:753–70. 10.1093/heapol/czae055 38938168 PMC11308613

[czag076-B12] Hoe C, Taber N, Champagne S et al Drink, but don't drive? The alcohol industry’s involvement in global road safety. Health Policy Plan 2021;35:1328–38. 10.1093/heapol/czaa097 33221890 PMC7886444

[czag076-B13] Iemmi V . Establishing political priority for global mental health: a qualitative policy analysis. Health Policy Plan 2022;37:1012–24. 10.1093/heapol/czac046 35763373 PMC9384251

[czag076-B14] Ionescu A, Mannell J, Vaughan M et al Misunderstood and underappreciated: a critical review of mental health advocacy and activism in low-and middle-income countries. Health Policy Plan 2024;39:528–39. 10.1093/heapol/czae016 38441280 PMC11095268

[czag076-B15] Jassat W, Moshabela M, Schneider H. Actor sensemaking and its role in implementation of the decentralized drug-resistant tuberculosis policy in South Africa. Health Policy Plan 2025;40:183–93. 10.1093/heapol/czae105 39506555 PMC11800982

[czag076-B16] Koon AD, Hawkins B, Mayhew SH. Framing and the health policy process: a scoping review. Health Policy Plan 2016;31:801–16. 10.1093/heapol/czv128 26873903 PMC4916318

[czag076-B17] Lencucha R, Moyo T, Labonte R et al Shifting from tobacco growing to alternatives in Malawi? A qualitative analysis of policy and perspectives. Health Policy Plan 2020;35:810–8. 10.1093/heapol/czaa057 32525201 PMC8060983

[czag076-B18] Mulcahy G, Boelsen-Robinson T, Hart AC et al A comparative policy analysis of the adoption and implementation of sugar-sweetened beverage taxes (2016–19) in 16 countries. Health Policy Plan 2022;37:543–64. 10.1093/heapol/czac004 35244693 PMC9113088

[czag076-B19] Namugumya BS, Candel JJ, Termeer CJ et al The framing of malnutrition by parliamentarians in Uganda. Health Policy Plan 2021;36:585–93. 10.1093/heapol/czab009 33709155

[czag076-B20] Novignon J, Lanko C, Arthur E. Political economy and the pursuit of universal health coverage in Ghana: a case study of the National Health Insurance Scheme. Health Policy Plan 2021;36:i14–21. 10.1093/heapol/czab061 34849898 PMC8633650

[czag076-B21] Parashar R, Gawde N, Gupt A et al Unpacking the implementation blackbox using' actor interface analysis’: how did actor relations and practices of power influence delivery of a free entitlement health policy in India? Health Policy Plan 2020;35:ii74–83. 10.1093/heapol/czaa125 33156935 PMC7646725

[czag076-B22] Parkhurst J . A social, not a natural science: engaging with broader fields in health policy analysis; comment on “modelling the health policy process: one size fits all or horses for courses?”. Int J Health Policy Manag 2023;12:. 10.34172/ijhpm.2023.8101 PMC10461874 37579369

[czag076-B23] Parkhurst J, Ghilardi L, Webster J et al Competing interests, clashing ideas and institutionalizing influence: insights into the political economy of malaria control from seven African countries. Health Policy Plan 2021;36:35–44. 10.1093/heapol/czaa166 33319225 PMC7938496

[czag076-B24] Ridde V, Caffin J-H, Hane F. External influences over Senegalese health financing policy: delaying universal health coverage? Health Policy Plan 2024;39:80–3. 10.1093/heapol/czad108 38011666 PMC10775215

[czag076-B25] Sabey CS . Implementation of mental health policies and reform in post-conflict countries: the case of post-genocide Rwanda. Health Policy Plan 2022;37:1248–56. 10.1093/heapol/czac074 36062976

[czag076-B26] Shawar YR, Crane LG. Generating global political priority for urban health: the role of the urban health epistemic community. Health Policy Plan 2017;32:1161–73. 10.1093/heapol/czx065 28582532 PMC5886225

[czag076-B27] Smith SL, Parashar R, Nanda S et al Shifting patterns and competing explanations for infectious disease priority in global health agenda setting arenas. Health Policy Plan 2024;39:805–18. 10.1093/heapol/czae035 38753344 PMC11384117

[czag076-B28] Sparkes SP, Bump JB, Özçelik EA et al Political economy analysis for health financing reform. Health Syst Reform 2019;5:183–94. 10.1080/23288604.2019.1633874 31369319

[czag076-B29] Sparkes SP, Shroff ZC, Hanson K. Still rethinking external assistance for health. Health Policy Plan 2024;39:i1–3. 10.1093/heapol/czad103 PMC10977908 38253448

[czag076-B30] Sriram V, Topp SM, Schaaf M et al 10 best resources on power in health policy and systems in low- and middle-income countries. Health Policy Plan 2018;33:611–21. 10.1093/heapol/czy008 29471544

[czag076-B31] Srivastava S, Bertone MP, Parmar D et al The genesis of the PM-JAY health insurance scheme in India: technical and political elements influencing a national reform towards universal health coverage. Health Policy Plan 2023;38:862–75. 10.1093/heapol/czad045 37436821

[czag076-B32] Tele A, Nyamai D, Shawar YR et al The political economy of adolescent mental health in Kenya. Health Policy Plan 2025;40:1017–26. 10.1093/heapol/czaf057 40879760 PMC12605744

[czag076-B33] Udokanma EE, Ogamba I, Ilo C. A health policy analysis of the implementation of the National Tobacco Control Act in Nigeria. Health Policy Plan 2021;36:484–92. 10.1093/heapol/czaa175 33393594

[czag076-B34] Walt G, Gilson L. Reforming the health sector in developing countries: the central role of policy analysis. Health Policy Plan 1994;9:353–70. 10.1093/heapol/9.4.353 10139469

[czag076-B35] Walt G, Shiffman J, Schneider H et al Doing’ health policy analysis: methodological and conceptual reflections and challenges. Health Policy Plan 2008;23:308–17. 10.1093/heapol/czn024 18701552 PMC2515406

[czag076-B36] Webster J, Hoyt J, Diarra S et al Adoption of evidence-based global policies at the national level: intermittent preventive treatment for malaria in pregnancy and first trimester treatment in Kenya, Malawi, Mali and The Gambia. Health Policy Plan 2021;35:1364–75. 10.1093/heapol/czaa132 33179027 PMC7886437

[czag076-B37] Whyle EB, Gilson L, Shroff Z. Developments in the field of Health Policy Analysis in low- and middle-income countries: a review of published literature 2008–2023. Health Policy Plan 2026. 10.1093/heapol/czag079 PMC13586625 42348916

[czag076-B38] Wireko I, Béland D, Kpessa-Whyte M. Self-undermining policy feedback and the creation of National Health Insurance in Ghana. Health Policy Plan 2020;35:1150–8. 10.1093/heapol/czaa080 32989440

